# A randomized sham-controlled trial on the effect of continuous positive airway pressure treatment on gait control in severe obstructive sleep apnea patients

**DOI:** 10.1038/s41598-021-88642-5

**Published:** 2021-04-29

**Authors:** Sébastien Baillieul, Bernard Wuyam, Dominic Pérennou, Renaud Tamisier, Sébastien Bailly, Meriem Benmerad, Céline Piscicelli, Thibault Le Roux-Mallouf, Samuel Vergès, Jean-Louis Pépin

**Affiliations:** 1 HP2 Laboratory, U1300, INSERM, Grenoble Alpes University, Grenoble, France; 2 Pôle Thorax et Vaisseaux, Grenoble Alpes University Hospital, Grenoble, France; 3 LPNC Laboratory (UMPR CNRS 5105), Grenoble Alpes University, Grenoble, France; 4 PMR Department, Grenoble Alpes University Hospital, Grenoble, France; 5 Laboratoire EFCR, CHU de Grenoble, CS 10217, 38043 Grenoble Cedex 09, France

**Keywords:** Hypoxia, Sleep disorders, Randomized controlled trials

## Abstract

To determine the effect of continuous positive airway pressure (CPAP), the gold standard treatment for obstructive sleep apnea syndrome (OSAS), on gait control in severe OSAS patients. We conducted a randomized, double-blind, parallel-group, sham-controlled monocentric study in Grenoble Alpes University Hospital, France. Gait parameters were recorded under single and dual-task conditions using a visuo-verbal cognitive task (Stroop test), before and after the 8-week intervention period. Stride-time variability, a marker of gait control, was the primary study endpoint. Changes in the determinants of gait control were the main secondary outcomes. ClinicalTrials.gov Identifier: (NCT02345694). 24 patients [median (Q1; Q3)]: age: 59.5 (46.3; 66.8) years, 87.5% male, body mass index: 28.2 (24.7; 29.8) kg. m^−2^, apnea–hypopnea index: 51.6 (35.0; 61.4) events/h were randomized to be treated by effective CPAP (n = 12) or by sham-CPAP (n = 12). A complete case analysis was performed, using a mixed linear regression model. CPAP elicited no significant improvement in stride-time variability compared to sham-CPAP. No difference was found regarding the determinants of gait control. This study is the first RCT to investigate the effects of CPAP on gait control. Eight weeks of CPAP treatment did not improve gait control in severe non-obese OSAS patients. These results substantiate the complex OSAS-neurocognitive function relationship.

## Introduction

Obstructive sleep apnea syndrome (OSAS) is one of the most prevalent chronic diseases affecting nearly 1 billion adults aged 30–69 years worldwide^1,2^. Chronic intermittent hypoxia and sleep fragmentation, resulting in systemic low-grade inflammation and oxidative stress, in time produce neural damage and cerebral homeostatic and neurovascular changes underlying the detrimental consequences of severe OSAS on cerebral structure and function^3,4^. OSAS-related neurocognitive impairment extensively affects attention and vigilance domains as well as executive functioning, which in turn impact everyday functioning, work performance and productivity^5–7^. Recently, gait abnormalities have been highlighted in severe OSAS^8–11^, with a dose–response relationship between OSAS severity and the magnitude of gait impairment^8^. Rather than a completely automatic task, gait has to be considered as a cognitively-demanding task, requiring attention and executive functions integrity^12^. Defects in these neurocognitive domains can be revealed and/or documented by gait abnormalities^13^. While the beneficial effects of continuous positive airway pressure (CPAP, the gold standard treatment for OSAS) on excessive daytime sleepiness and everyday functioning is well established^14^, its ability to reverse neurocognitive impairment remains debated, highlighting a complex OSAS-neurocognitive relationship^15–17^. To date, one open-labelled^9^ and one non-randomized controlled study^10^ have shown positive effects of CPAP treatment on gait control in OSAS patients. To validate these previously described effects a randomized, controlled (effective vs. sham-CPAP, the acknowledged CPAP placebo), double-blind trial was lacking. Furthermore, neurophysiological measurements are required to determine the cerebral correlates of the potential CPAP-induced changes in gait control. The main objective of the present parallel randomized controlled trial (RCT) was to investigate the impact of an 8-week CPAP treatment on gait control evaluated by stride time variability (STV) in severe OSAS patients, compared to sham-CPAP. We hypothesized that: (1) gait control in severe non-obese OSAS patients would be improved by CPAP treatment and (2) those improvements might be paralleled by changes in the determinants of gait control, i.e. postural control, cognitive performance and cerebral oxygenation assessed by functional near-infrared spectroscopy (fNIRS) while walking.

## Results

### Patients characteristics

**Figure 1:**
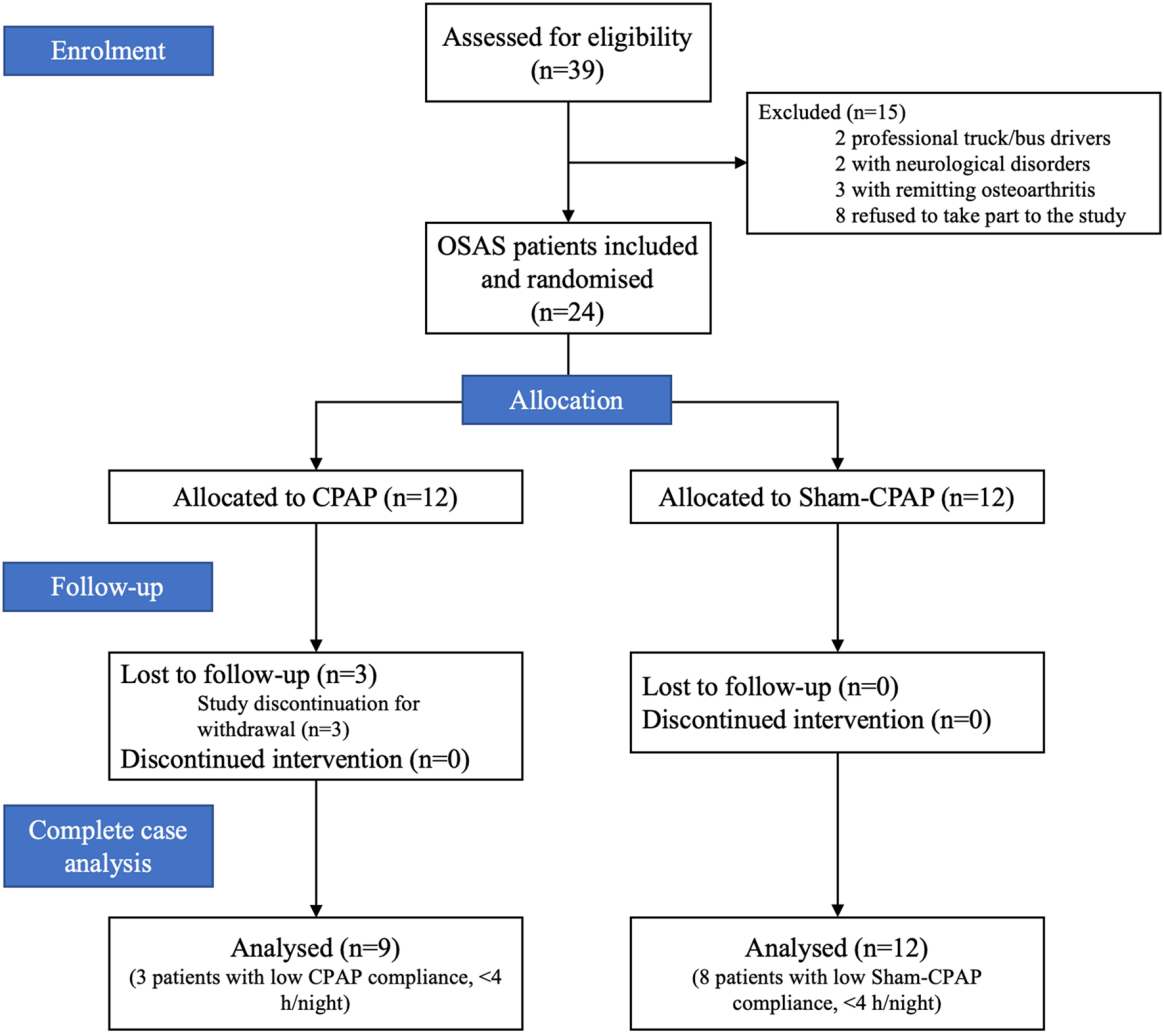
Study flow chart. *CPAP* continuous positive airway pressure treatment, *OSAS* obstructive sleep apnea syndrome.

Twenty-four severe OSAS patients were included and randomized to be treated by effective CPAP (n = 12) or by sham-CPAP (n = 12). Twenty-one patients completed all the evaluations, as three patients in the effective CPAP group withdrew, due to personal constraints (Fig. 1). Patient characteristics are shown in Table 1. There was no significant difference regarding all baseline anthropometric and sleep apnea characteristics. Three out of nine patients (33.3%) in the CPAP group and eight out of 12 patients (66.7%) in the sham-CPAP group showed low compliance (< 4 h of use per night) and the percentage of nights with CPAP usage > 4 h/night was significantly lower in the sham-CPAP group (*p* = 0.02). Patients in the CPAP group were effectively treated, as shown by them achieving normal values for residual AHI (Table 1).

**Table 1:** Participants characteristics, obstructive sleep apneas characteristics at baseline and after 8 weeks of CPAP or Sham-CPAP.

	All participants	Sham-CPAP	CPAP
**Baseline characteristics**			
No. of participants (n)	24	12	12
Age (years)	59.5 [46.3; 66.8]	58.4 [46.5; 67.7]	60.4 [42.2; 65.6]
Male sex, n (%)	21 (87.5)	11 (91.7)	10 (83.3)
Education (years)	11.5 [11.0; 14.0]	12.0 [11.0; 17.0]	11.0 [11.0; 13.5]
BMI (kg m^−2^)	28.2 [24.8; 29.8]	28.7 [24.6; 29.7]	28 [25.3; 30.7]
Smoking, n (%)	9 (37.5)	3 (25.0)	6 (50.0)
Past cardiovascular events, n (%)	3 (12.5)	2 (16.7)	1 (8.3)
Systemic hypertension, n (%)	9 (37.5)	5 (41.7)	4 (33.3)
Diabetes, n (%)	2 (8.3)	2 (16.7)	0 (0.0)
Dyslipidemia, n (%)	3 (12.5)	2 (16.7)	1 (8.3)
MMSE Score (/30)	28.0 [25.0; 29.0]	27.5 [25.5; 29.0]	28.0 [27.0; 28.0]
ESS Score (/32)	11.5 [8.0; 14.5]	11.5 [9.0; 13.0]	11 [7.5; 15.5]
**Sleep studies**			
AHI (events h^−1^)	51.6 [35.0; 61.4]	38.1 [32.7; 59.7]	54.4 [46.4; 62.0]
Apnea index (events h^−1^)	15.0 [7.9; 40.9]	11.7 [7.0; 15.0]	21.6 [11.6; 43.7]
Hypopnea index (events h^−1^)	25.3 [21.2; 38.8]	23.9 [21.2; 42.7]	28.0 [18.9; 35.9]
ODI (events h^−1^)	38.8 [22.5; 51.9]	28.5 [18.5; 49]	44.1 [36.2; 53.3]
Mean nocturnal SpO_2_ (%)	93.0 [91.5; 94]	93.2 [92.5; 95]	92.4 [90.8; 94]
SpO_2_ nadir (%)	81.0 [72.0; 83.0]	81.0 [76.0; 84.0]	78.5 [71.5; 83.0]
SpO_2_ < 90% (% of recording time)	5.3 [1.8; 12.0]	2.0 [1.0; 11.7]	5.7 [3.6; 25.5]
**Post intervention**			
No. of participants (n)	21	12	9
BMI (kg m^2^)	28.3 [24.9; 29.9]	28.5 [25.0; 29.9]	27.7 [24.9; 29.9]
ESS Score (/32)	6.0 [4.0. 10.0]	7.5 [4.3; 12.0]	5.0 [2.5; 9.5]
Mean CPAP compliance (h night^−1^)	3.8 [2.5; 5.8]	3.5 [0.0; 6.3]	4.7 [3.8; 5.3]
Percentage of nights with CPAP usage > 4 h/night (%)	37.5 [0.0; 70.0]	0.0 [0.0; 41.5]	66.0 [37.5; 80.0]
Residual AHI (events h^−1^)	–	–	1.7 [1.2; 2.0]

*AHI* apnea–hypopnea index, *BMI* body mass index, *CPAP* continuous positive airway pressure, *ESS* Epworth Sleepiness Scale, *MMSE* Mini Mental State Examination, *ODI* oxygen desaturation index, defined as a drop in SpO_2_ > 3% for at least 10 s ; SpO_2_: pulsed dioxygen saturation; SpO_2_ < 90%: percentage of recording time spent at a SpO_2_ < 90%

Past cardiovascular events: defined as a past history of non-fatal stroke, non-fatal myocardial infarction, transient ischemic attack, any cardiac event of coronary origin (including revascularization procedures), hospitalizations due to cardiac or cerebrovascular causes (acute coronary syndrome, stroke, heart failure, heart rhythm disorder including atrial fibrillation, bleeding, etc.).

AHI Flow was measured using the CPAP machine’s internal microprocessor.

Data are presented as median [Q1; Q3] or as number (%) of participants.

### Primary outcome: stride time variability

**Figure 2:**
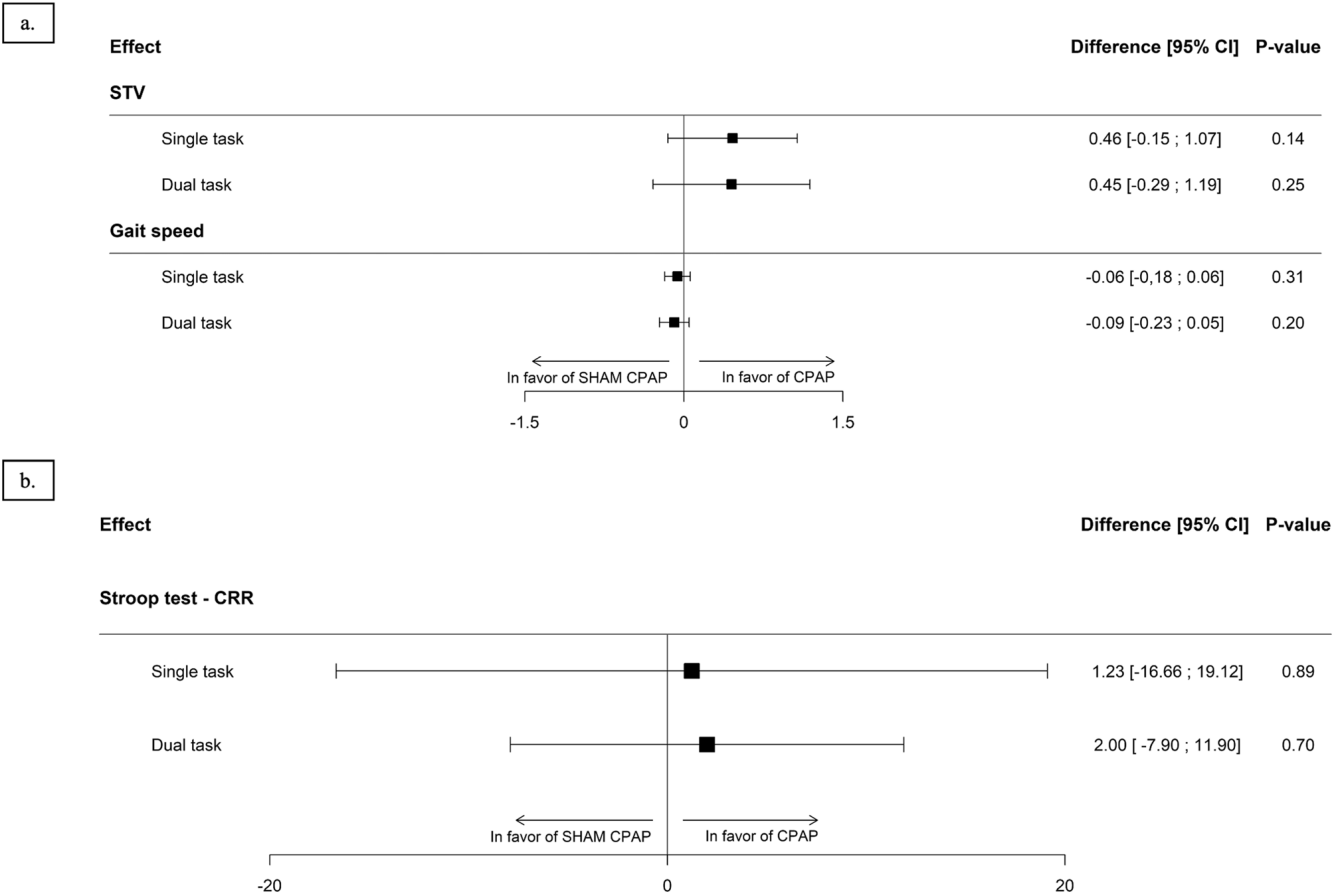
Effect of continuous positive airway pressure (CPAP) on gait control (**a**) and Stroop test performance (**b**) assessed in single (gait or Stroop test only) and dual task (gait and Stroop test performed simultaneously) (linear mixed effect model analysis). *CPAP* continuous positive airway pressure, *CRR* correct response rate, *DT* dual task, *ST* single task, *STV* stride time variability.

There was no significant difference between the CPAP and sham-CPAP groups for the primary outcome, STV, both under ST [Group effect β (Standard Error (SE)) = 0.46 (0.31), *p* = 0.14; Period effect β (SE) = −0.09 (0.13), *p* = 0.50] and DT [Group effect β (SE) = 0.45 (0.38), *p* = 0.25; Period effect β (SE) = −0.26 (0.09) *p* < 0.01] (Fig. 2 a. and Supplementary Table S1).

### Overground gait parameters and dual-task gait performance

Gait parameters did not differ between the two groups at baseline and CPAP elicited no significant improvement in gait parameters under either ST or DT conditions (Supplementary Table S1). Effective CPAP produced no significant change in gait speed (Fig. 2a). Cognitive performance in the Stroop Test, evaluated by the CRR, was not improved by CPAP treatment in both ST and DT (Fig. 2b).

### Cognitive performance

Results of the neurocognitive evaluations are presented in Table 2. There was no significant difference between the two groups at baseline. The only significant improvement observed in the effective CPAP group after the 8 weeks of intervention was for the Stroop Interference test (*p* = 0.02).

**Table 2:** Results of the neuropsychological assessments and impact of CPAP treatment.

Cognitive domain and test	Pre-intervention			Post-intervention			Comparison between pre and post-intervention *p *values	
	Sham-CPAP	CPAP	*p*	Sham-CPAP	CPAP	*p*	Sham-CPAP	CPAP
**Global cognitive assessment**								
MMSE (Total score /30)	27.5 [25.5; 29.0]	28.0 [27.0; 28.0]	0.73	28.0 [27.0; 29.0]	27.0 [11.5; 28.5]	0.18	0.59	0.08
**Memory**								
16-item free and cued recall test								
Free recall (/48)	25.5 [23.5; 27.5]	28.5 [25.0; 30.5]	0.13	30.5 [26.0; 32.0]	31.0 [28.0; 32.0]	0.78	**0.04**	0.75
Delayed free recall (/16)	10.0 [9.0; 12.5]	11.0 [10.0; 12.5]	0.60	12.0 [10.0; 12.0]	10.0 [9.0; 12.0]	0.18	0.17	0.41
Total recall (/48)	44.5 [43.0; 46.5]	46.5 [44.5; 47.5]	0.16	46.0 [44.5; 47.0]	46.0 [42.0; 47.0]	0.72	0.14	0.27
Delayed total recall (/16)	16.0 [15.0; 16.0]	16.0 [16.0; 16.0]	0.26	16.0 [15.0; 16.0]	15.0 [15.0; 16.0]	0.53	1.00	0.06
**Executive functions**								
PASAT								
Number of errors—1st third	3.0 [2.0; 5.0]	4.0 [2.0; 6.0]	0.45	1.0 [0.0; 3.0]	4.0 [0.0; 7.0]	0.42	**0.04**	0.13
Number of errors—2nd third	4.0 [2.0; 6.0]	5.0 [3.0; 7.0]	0.73	1.0 [0.0; 4.0]	6.0 [4.0; 9.0]	**0.03**	0.20	0.25
Number of errors—3rd third	2.0 [1.0; 6.0]	5.0 [3.0; 9.0]	0.21	1.0 [0.0; 4.0]	4.0 [3.0; 8.0]	**0.03**	**0.02**	0.50
Total correct answers (/60)	48 [45; 56]	45 [38 ; 49]	0.37	57 [53 ; 58]	48 [37 ; 51]	**0.03**	**0.02**	0.19
Stroop test								
CRR—denomination	215.1 [167.1; 238.1]	212.9 [192.6; 245.2]	0.71	208.3 [156.3; 238.1]	208.3 [192.3; 222.2]	0.88	0.49	0.30
CRR—reading	166.7 [117.3; 186.2]	158.4 [146.4; 198.5]	0.61	172.4 [124.4; 208.3]	169.5 [161.3; 188.7]	0.85	0.24	0.25
CRR—interference	82.2 [60.6; 103.3]	75 [67.7; 90.4]	0.84	82.6 [68.5; 107.5]	98 [89.3; 101.1]	0.41	0.15	**0.02**
Stroop interference time	215.7 [108.9; 306.1]	154.5 [126.1; 218.8]	0.55	186.8 [149.3; 344.8]	227.3 [204.1; 285.7]	0.55	0.28	0.10
Trail making test								
TMT A—number of errors	0.0 [0.0; 0.5]	0.0 [0.0; 0.0]	0.60	0.0 [0.0; 1.0]	0.0 [0.0; 1.0]	1.00	1.00	1.00
TMT A—time (S)	29.0 [25.5; 37.5]	36.5 [28.5; 40.5]	0.35	30.5 [27.0; 35.0]	33.0 [30.0; 37.0]	0.58	0.56	0.52
TMT B—error	0.5 [0.0; 3.0]	0.0 [0.0; 0.0]	0.13	0.0 [0.0; 0.0]	0.0 [0.0; 1.0]	0.10	0.06	0.63
TMT B—time (s)	76.0 [68.5; 110.0]	85.5 [67.5; 92.5]	0.89	79.5 [55.0; 106.0]	130.0 [71.0; 142.0]	0.38	0.29	0.07
TMT B-A—number of errors	0.0 [0.0; 2.0]	0.0 [0.0; 0.0]	0.48	0.0 [-1.0; 0.0]	0.0 [-0.5; 1.0]	0.33	0.18	1.00
TMT B-A—time (s)	48.5 [36.5; 74.0]	46.5 [37.0; 59.0]	0.65	48.5 [30.5; 68.0]	42.0 [8.5; 104.0]	0.82	0.32	0.72
Wechsler Adult Intelligence Scale IV (WAIS IV)								
Memory—digit span forward	5.5 [5.0; 6.0]	5.5 [4.0; 7.0]	1.00	6.0 [5.0; 7.0]	5.0 [5.0; 5.0]	0.09	0.13	0.50
Memory—digit span backward	4.0 [3.0; 4.0]	4.0 [3.0; 4.5]	0.56	4.0 [3.0; 5.0]	4.0 [3.0; 4.0]	0.46	0.11	0.63
Memory—standard note (/19)	8.5 [7.0; 11.0]	9.0 [7.5; 10.0]	0.89	11.0 [8.0; 12.0]	7.5 [7.0; 9.5.0]	0.10	**0.04**	0.50
Code—standard note (/19)	10.0 [6.5; 11.5]	9.5 [7.0; 11.0]	0.98	11.0 [7.0; 13.0]	8.0 [8.0; 11.5]	0.44	**0.03**	0.28
Tower of Hanoï								
Index Score	11.0 [10.0; 12.0]	12.0 [10.0; 13.0]	0.45	12.0 [10.0; 13.0]	10.0 [10.0; 12.0]	0.14	0.30	0.29

*CPAP* continuous positive airway pressure, *CRR* correct response rate, *MMSE* mini mental state examination, *PASAT* paced auditory serial addition test, *TMT* trail making test; *WAIS* Wechsler Adult Intelligence Scale.

Analysis: Data are presented as median [Q1; Q3]. Analysis of data by Wilcoxon–Mann–Whitney test for continuous data and by a χ^2^ or Fisher exact test for categorical data. Significant results are displayed in bold.

MMSE: the displayed result is the total score of the test, with a maximum score of 30, higher score indicating better global cognitive functioning.

16-item free and total recall: for the free and total recall tests, results are presented as the sum of the three consecutive trials, with a maximum score of 48. For the delayed free and total recall tests, maximum score is 16, higher score indicating better performance.

Paced Auditory Serial Addition test: A pre-recorded tape delivered a random series of 61 numbers from 1 to 9, at a constant rate of 1 number every 4 s. Participants were instructed to add pairs of numbers such that each number was added to the one that immediately preceded it on the recording: the second was added to the first, the third to the second, the fourth to the third, and so on. The response had to be given before the presentation of the next stimulus (4 s later). The sum of any given pair never exceeded 15. The number of correct responses was recorded (PASAT maximum = 60).

Stroop test: The test is composed of 3 different parts: (1) Denomination: participants have to name 48 colored patches (2) Reading: participants have to read 48 color names, written in a congruent color (The word “Red” written in red color font) and (3) Interference: task requiring response inhibition. Participants have to give the color font of 48 color names, written in an incongruent color (The word “Blue” written in green color font; correct answer: green). For each part of the test, time and number of errors are recorded and account for task performance. The displayed results are the correct response rates (CRR = Response rate per second × Percentage of correct responses) for the three parts of the test. Higher CRRs indicate better performance. Stroop interference time = Time for Stroop Interference test (s) − (Time for Stroop Denomination test (s) + Time for Stroop reading test (s))/2. A low score (seconds) indicates better executive function.

Trail Making Test: the displayed results are the time and number of errors for each part of the test, as well as the difference between the time and number of errors between test B and A.

### Standing balance

There was no significant difference in postural control between the two groups at baseline (Supplementary Table S2). CPAP elicited no significant change in postural control evaluated by the CoP Area both in ST [Group effect β (SE) = −15.48 (27.88), *p* = 0.58; Period effect β (SE) = 0.37 (17.36) *p* = 0.98] and DT [Group effect β (SE) = −16.85 (22.60), *p* = 0.46; Period effect β (SE) = 26.22 (15.59) *p* = 0.11]. There was no significant improvement for all the other postural parameters following CPAP treatment (Supplementary Table S2). Cognitive performance at the Stroop Test, evaluated by the CRR, was not improved by CPAP treatment both in ST [Group effect β (SE) = 1.23 (9.13), *p* = 0.89; Period effect β (SE) = 10.31 (3.64) *p* = 0.01] and in DT [Group effect β (SE) = 2.62 (4.32), *p* = 0.55; Period effect β (SE) = 6.42 (1.71) *p* < 0.01].

### Treadmill dual-task gait and cerebral oxygenation

**Figure 3:**
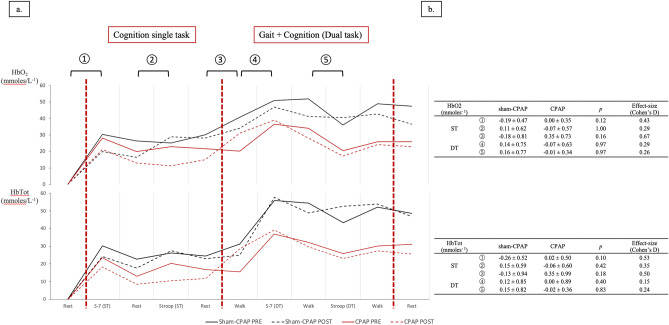
Evolution of prefrontal cortices Oxy[HbO2]- and Total[HbTot]-hemoglobin concentration assessed by functional Near Infrared Spectroscopy during the treadmill test. (**a**) Evolution of [HbO2] and [HbTot] (in mmoles^−1^) pre (continuous line) and post (dotted line) intervention for sham-CPAP (n = 11, represented in black) and CPAP (n = 6, represented in red) groups. Data are the measured mean values of [HbO2] and [HbTot] over the first 30-s of each task, averaged for left and right prefrontal cortices. To assess the evoked hemodynamic response, five activation indices were calculated as the difference in [HbO2] and [HbTot] between task and rest (in ST) or task and walk (in DT): ① S-7 ST; ② Stroop test ST; ③ Walk ST; ④ S-7 DT; ⑤ Stroop test DT. (**b**) Comparisons of delta pre-post intervention of [HbO2] and [HbTot] between sham-CPAP (n = 11) and CPAP (n = 6) groups. Data are mean ± 1 standard deviation. Analysis of data by Wilcoxon-Mann–Whitney test for continuous data, provided with Cohen’s D for effect size estimation. *CPAP* continuous positive airway pressure, *DT* dual task, *HbO2* Oxy-hemoglobin concentration, *HbTot* total hemoglobin concentration, *S-7* Serial S-7 tasks, *ST* single task.

Preferred walking speed at baseline did not differ between the two groups [median (Q1; Q3) gait speed = 3.00 (2.58; 3.30) in the sham-CPAP group vs*.* 2.85 (2.35; 3.38) in CPAP group, *p* = 0.56]. There was no significant difference between the two groups at baseline. After eight weeks of intervention, CPAP elicited no significant change in cognitive performance in either ST or DT (Supplementary Table S3). Regarding pre-frontal cortex hemodynamics assessed by fNIRS, 11 out of 12 patients (91.7%) in the sham-CPAP group and 6 out of 9 (66.7%) in the CPAP group had valid pre- and post-intervention recordings (*p* = 0.27). There was no significant difference regarding [HbO_2_], [HHb], [HbTot] and TSI between the two groups, pre and post intervention, despite higher baseline values in the sham-CPAP group (Fig. 3).

## Discussion

In this randomized, sham-controlled trial (RCT), eight weeks of CPAP treatment did not improved gait control of severe OSAS patients.

We found no improvement in STV, our primary outcome, following eight weeks of CPAP treatment. The STV values obtained at baseline were comparable to those of the severe OSAS patients reported by Celle et al.^8^ and in our previous study^10^ showing impaired gait control in OSAS patients. This negative result contrasts with that of two recent uncontrolled interventional studies. Allali et al.^9^ showed improvements in gait speed and temporal gait parameters specifically in dual tasks. Their study included mostly obese patients, lacked an appropriate control group and the changes in gait performance they reported were subtle with limited clinical relevance. In our previous prospective, non-randomized controlled study^10^, we showed that at baseline OSAS patients had significantly larger STV compared to matched healthy controls. After eight weeks of CPAP treatment, STV was significantly improved and no longer different from controls. The lack of an OSAS group treated by sham-CPAP and the absence of a retest of the controls eight weeks after baseline assessment limited the conclusions of this previous study. The present study, using a stronger methodological design, suggests that eight weeks of CPAP may be insufficient to reverse gait control impairments in severe OSAS patients. This result was supported by the lack of changes after CPAP treatment in the main determinants of gait control (i.e. brain oxygenation and cognitive performance). A key outcome was DT gait assessment using a Stroop test. We found no difference between the two groups but a mutual facilitation^18^ reflecting an improvement in DT performance both in gait (i.e. a reduction in STV) and cognition (i.e. an increased Stroop CRR) compared to ST performance. This might be explained by an exercise-induced arousal effect^19^, the positive incidence of acute physical activity on cognitive performance. However, such an effect is expected to occur only in patients with morphologically and functionally unaltered prefrontal cortices suggesting only slight neurological consequences of OSAS prior to treatment in the included patients^18^.

All patients underwent a comprehensive neurocognitive evaluation, covering the domains supposed to be most impaired in OSAS^5,20^. CPAP treatment elicited no significant change in cognitive performance, except an improvement in the Stroop Interference test. We had included patients without significant cognitive deficiency at baseline and most of them were highly educated, with at least half having more than 11 years of formal education. Thus, included patients were normal or supra normal, free of comorbidities, and with probably a high cognitive reserve^6^, representing a preserved adaptability of cognitive processes explaining their reduced susceptibility to cognitive deterioration with aging or pathologies^21^.

During cognitive or motor tasks, cerebral hemodynamic regulation is essential so as to deliver the adequate amount of oxygen and substrates required for brain metabolism^22^. Severe OSAS might have impaired cerebral hemodynamic regulation^23,24^. CPAP treatment, through suppression of nocturnal intermittent hypoxia and fragmented sleep, is thought to limit OSAS-related neuroinflammation and improve cerebrovascular hemodynamics^3^. CPAP has a clear acute impact in suppressing acute cerebral hemodynamic abnormalities occurring concurrently with sleep obstructive events^25^. Some studies have suggested a partial improvement in cerebral blood flow (CBF) in the frontal areas during wakefulness in OSAS patients with a dose–response effect-size related to CPAP adherence^26,27^. However, in our well controlled RCT no chronic impact of CPAP treatment on prefrontal cortices hemodynamics was found, as assessed by fNIRS during daytime walking. This result is consistent with those of a previous study from our group^28^, showing no improvement in prefrontal cortex hemodynamics while performing an incremental cycling test following eight weeks of CPAP treatment.

The major strength of our study is its randomized controlled design which is unique in the field of gait control and OSAS. We acknowledge that the duration of the CPAP intervention might not have been long enough for CPAP to have any effect. Although we note that most of the existing studies on neurocognitive function or brain imaging in OSAS patients have used similar timeframes. Moreover, one third of the patients of the CPAP group showed low CPAP compliance (< 4 h of use per night). Altogether, this may have dampened the expected positive effect of CPAP on gait control.

We included only non-obese OSAS patients. This choice was driven by the fact that obesity is a cofounding factor when assessing gait control and postural control, as overweight and even more obesity directly affect gait control and gait kinetics^29,30^. Another limitation could be that the gait and postural tasks might have been too easy for participants, with parameters already satisfactory at baseline, which reduced the chances to conclude. Lastly, the small sample size of highly selected patients, together with three loss of follow-up patients in the CPAP group may have impacted our results. As we included only severe OSAS patients, further generalization of the study findings should be restricted to OSAS patients with an AHI > 30 events. h^−1^, with a low burden of comorbidities.

## Conclusion

This first randomized controlled trial in the field showed that eight weeks of CPAP treatment did not improve gait control in severe non-obese OSAS patients. Long-term real-life observational data on different clusters of phenotypes^31^ might help to identify specific subgroups for whom CPAP could have a significant impact on gait control, which could then be tested in further RCTs.

## Methods

### Study design

This randomized, double-blind, parallel-group, sham-controlled study was monocentric, performed at Grenoble Alpes University Hospital, France. The study was approved by an independent Ethics Committee (CPP Sud-Est V, Grenoble, France, 14-CHUG-46, ID RCB: 2014-A01523-44, date of initial approval: November 12th, 2014); conducted in accordance with applicable good clinical practice requirements in Europe, French law and ethical principles of the Declaration of Helsinki; and is registered on the ClinicalTrials.gov site (NCT02345694; date of first registration 26/01/2015; study start date 03/02/2015). Written informed consent was obtained from all patients prior to their participation in the study. Informed consent was obtained to publish the images in an online open access publication.

An external data quality control was performed systematically for the following criteria: informed consent, complications, adverse events, and case report forms.

The primary study endpoint was the change in STV of OSAS patients after eight weeks of CPAP treatment, in comparison with sham-CPAP treatment.

### Participants

Patients were recruited from a consecutive sample at the Sleep Laboratory of Grenoble Alpes University Hospital and from the Outpatient Sleep Clinic in a French tertiary-care university hospital (Grenoble, France) between February 2015 and December 2018. Inclusion criteria were: (1) age ≥ 18 years and < 70 years, (2) non-obese patients (body-mass index (BMI) < 30 kg. m^−2^), to control for obesity-related gait kinetic changes^29^ (3) severe OSAS (apnea–hypopnea index, AHI > 30. events h^−1^) on polysomnography or respiratory polygraphy, (4) to be naive of CPAP treatment and (5) to present with a strictly normal neurological examination. Patients were not included if they declined to participate or were unable to give their informed consent. Patients with any of the following criteria were also not included: (1) the presence of any medical condition supposed to interfere with gait, (2) cognitive impairment, defined as a score on the Mini-Mental State Examination (MMSE^32^) < 24/30, (3) current pregnancy, (4) ongoing hypnotic or central nervous system targeted medication, (5) chronic alcohol consumption and (6) a profession requiring an effective CPAP treatment (*e.g.* public transport or truck drivers).

### Overnight sleep studies

Obstructive sleep apnea syndrome (OSAS) diagnosis was based on an overnight sleep study (respiratory polygraphy or polysomnography), performed before inclusion and treatment by continuous positive airway pressure (CPAP) or sham-CPAP according to standard procedures^33^. Polysomnographic recordings were undertaken with electroencephalography (EEG) electrode positions C3/A2-C4/A1-Cz/01 of the international 10–20 Electrode Placement System, eye movements, chin electromyogram and ECG with a modified V2 lead. Sleep was scored manually according to standard criteria. For polysomnographic and polygraphic recordings, airflow was measured with nasal pressure prongs, together with the sum of oral and nasal thermistor signals. Respiratory effort was monitored using abdominal and thoracic bands. Oxygen saturation was measured using a pulse oximeter and oxygen desaturation index (ODI), mean nocturnal SpO_2_, and percentage of recording time spent at a SpO_2_ < 90% were also calculated. Respiratory events were scored manually by a trained sleep specialist and the apnea–hypopnea index (AHI) was calculated from the number of apneas and hypopneas per hour of sleep according to international guidelines^34^. An apnea was defined as the complete cessation or a reduction of at least 90% of airflow for at least 10 s and hypopnea as a reduction of at least 30% in the nasal pressure signal associated with either oxygen desaturation of at least 3% or an EEG arousal, both lasting for at least 10 s^35^. Apnea was classified as obstructive, central or mixed, according to the presence or absence of respiratory efforts. The classification of hypopnea as obstructive or central was based on the thoraco-abdominal band signal and the shape of the respiratory nasal pressure curve (flow limited aspect or not). Severe OSAS was defined as an AHI > 30 events h^−1^.

### Evaluation protocol

At baseline and at the end of the 8-week interventional period the same parameters were recorded following the same protocol for evaluation, performed in the same order at the same hour of the day: (1) baseline clinical assessment, (2) single task (ST) and dual task (DT) overground gait assessments, using a dual-task paradigm similar to our previous work^10^, (3) comprehensive neuropsychological assessment, (4) postural control assessment during ST and DT, using a dual-task paradigm similar to our previous work^10^ and (5) treadmill gait assessment in ST and DT, with continuous recording of prefrontal cortex functional activation using functional Near-Infrared Spectroscopy (fNIRS). Following the collection of baseline parameters patients were randomized to be treated by CPAP or sham-CPAP for eight weeks (the duration of interventions previously used in studies investigating the effects of CPAP treatment on cognition^15^ and gait^9,10^). Patients assigned to the CPAP group were equipped with an auto-titrating device (Autoset Spirit, ResMed, UK or Remstar Auto, Philips Respironics, Murrysville, PA, USA) provided by a home care provider (Agir à Dom, France). Pressure was homogenously set between 6 and 14 cmH_2_O in the effective CPAP group. Patients receiving sham-CPAP had a similar device, delivering a pressure that was too low to suppress sleep respiratory events, as previously validated^36–38^. As a double-blind protocol, investigators and the study team as well as patients were blinded to treatment allocation. CPAP compliance and residual AHI^39^ under CPAP were downloaded from the CPAP device software.

#### Baseline clinical assessment

Prior to all gait and postural assessments, patients underwent a screening history and physical examination to ensure that they were free of significant orthopedic, neurological and visual disorder which could interfere with the outcomes of the present study. Cardiovascular risk factors were recorded, including past or actual smoking, systemic hypertension, diabetes and dyslipidemia (Table 1). Past cardiovascular events, including non-fatal stroke and non-fatal myocardial infarction, transient ischemic attack, any cardiac event of coronary origin (including vascular recanalization procedures), hospitalization due to cardiac or cerebrovascular causes (acute coronary syndrome, stroke, heart failure, heart rhythm disorder including atrial fibrillation, bleeding, etc.) were retrieved from medical interviews. Patients were then tested for their ability to distinguish the four colors (red, yellow, blue, green) used in our Stroop test appropriately by using the same screen setting as during the dual-task assessments. They completed an Epworth Sleepiness Scale (ESS).

#### Overground gait assessment

**Figure 4:**
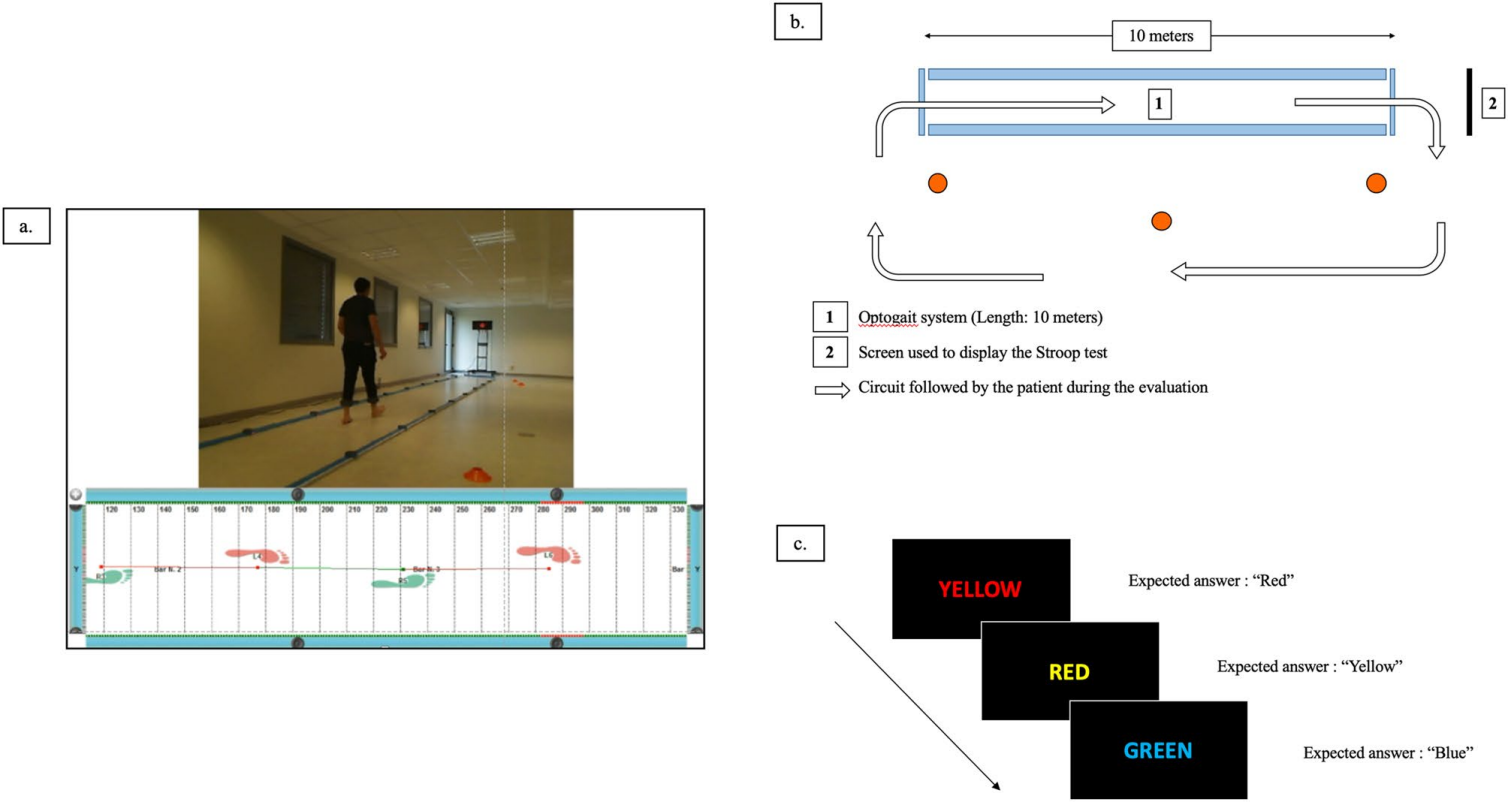
Overground gait and stride time variability assessment experimental setting. (**a**) Picture of the experimental setting. (**b**) Schematic representation of the oval gait circuit (outward in the 10-m long corridor delineated by the Optogait system, return outside of the corridor). Stroop test was displayed on a black background screen installed at the end of the corridor. (**c**) Schematic representation of the visuo-verbal Stroop test. Patients were instructed to name the words font color and to inhibit reading the word (Correct answers here: “red” then “yellow” then “blue”). Words were presented one by one, and the evaluator skipped manually to the next one after the subject gave an oral response.

Spatiotemporal gait parameters were recorded using a modular optoelectronic floor-based system (OptoGait, Microgate, Bolzano, Italy), which consists of two parallel 1-m bars (one emitting, containing 96 lights diodes and one receiving bar). The system demonstrated high reliability for the assessment of spatiotemporal gait parameters^40^. Ten emitting and ten receiving bars were disposed parallel to each other to build a 10-m long, 1.5-m width walkway. Patients walked barefoot, at their self-selected comfortable speed, continuously around an oval circuit^10^ (Fig. 4a, b). Each participant completed three familiarization loops prior to the 20 evaluation loops, alternating ST (10 evaluation loops, gait only) and DT (10 evaluation loops, gait and Stroop test performed simultaneously). Gait kinetic parameters were recorded at 1 kHz sample frequency and analyzed using the OptoGait software (version 1.10.7.0, Microgate). The average value of all recorded steps was used for data analysis. The following gait kinetic parameters were recorded and calculated: speed (m.s^−1^), cadence (step.s^−1^), stride time (s), total double support (percentage of total gait cycle time), step width (cm). The walk ratio (WR, cm/(steps/min))^41^, a speed-independent index of the overall neuromotor gait control, which reflects balance, between-step variability, and attentional demand was calculated as follows: WR = Step Length (cm)/cadence (steps/min).

Stride time variability (STV), our primary study endpoint, is a marker of gait control related to cerebral integrity and greater STV values reflect impaired gait control^42^. STV was calculated as the mean of the coefficient of variation of stride time as follows^42^:

$$ {\text{Coefficient}}\,{\text{of}}\,{\text{Variation}}\,{\text{of}}\,{\text{Stride}}\,{\text{Time}} = {\text{Standard}}\,{\text{Deviationof}}\,{\text{Stride}}\,{\text{Time/Mean}}\,{\text{Stride}}\,{\text{Time}} \times 100. $$

#### Dual-task paradigm

The DT paradigm consists in the assessment of the interferences occurring when a motor and a cognitive task are performed simultaneously^12,43^. This can result in performance decrements in one or both of the tasks, suggesting the simultaneous engagement of the same functional brain subsystems^44^. We used the same DT paradigm as in our previous work^10^, combining gait and postural assessments with a Stroop color-word interference test (Fig. 4c), a cognitive task considered to specifically assess executive functions. Stroop test consists in color names (blue, red, green and yellow) written in a conflicting font color. Participants were instructed to name the word font color and to inhibit reading the word (e.g., the word “green” written in red font color). To avoid learning effects, we used 30 different versions of the Stroop test, presented randomly to the participants throughout the different assessments. The Stroop test was displayed on a black background screen installed at the end of the corridor, which height was adjusted for each participant and for each evaluation. Words were presented one by one, and the evaluator skipped manually to the next one after the subject gave an oral response. The number of correct answers and errors were recorded by a trained evaluator. In ST, a red sight was displayed on the black background screen. In DT, participants were asked to perform both the motor and cognitive tasks at the best of their capacity without any task prioritization. The correct response rate for Stroop test performance in ST and DT gait assessments^45^ was calculated as follows:

$$ {\text{Correct}}\,{\text{response}}\,{\text{rate}}\,\left( {{\text{CRR}}} \right) \, = {\text{ Response}}\,{\text{rate}}\,{\text{per}}\,{\text{second }} \times {\text{ Percentage}}\,{\text{of}}\,{\text{correct}}\,{\text{responses}}. $$

To deepen our understanding of the complex interactions between gait, posture and cognition, we calculated the dual task effect (DTE)^18^ for each gait, postural and cognitive parameter, as follows:

$$ {\text{DTE}} = \left| {\left( {{\text{DT}}\,{\text{parameter}}\,{\text{performance}} - {\text{ST}}\,{\text{postural}}\,{\text{performance}}} \right){\text{/ST}}\,{\text{postural}}\,{\text{performance}}*100} \right| $$

DTE quantifies the dual-task interference, i.e. the relative change in performance associated with dual-tasking. Indeed, as attention or executive functions are limited resources, dividing attention between two concurrent tasks can result in a decrement in performance in one or both of the tasks, relative to when each task is performed alone^18^. Therefore, the magnitude of DT interference is influenced by the interaction between the two tasks.

#### Neuropsychological assessment

A comprehensive neuropsychological assessment, covering the most frequently impaired domains in OSAS^5,20^ (i.e. episodic memory and executive functions) was administered by a certified neuropsychologist, blind for treatment allocation, always at the same time of the day, in the same order:

- Global cognitive function: MMSE
- Episodic memory: 16-item free and cued recall test
- Executive functions: processing speed and working memory (Code and digit span forward and backward, Wechsler Adult Intelligence Scale IV), sustained attention (Paced Auditory Serial Addition test), visuomotor speed (Trail making test A), mental shifting (Trail making test B), interference and inhibition (Stroop test), planification skills (Tower of Hanoï).

Neuropsychological assessment lasted approximatively 1 h, with short breaks between each test to avoid fatigue.

Raw performances at the different evaluations are displayed in Table 2.

#### Standing posture

Posture and gait are interrelated functions and their control is anatomically and functionally intertwined^46^. Moreover, postural control is altered in OSAS^47^. We assessed standing balance using a posturographic platform (Feetest 6, TechnoConcept, Céreste, France), composed of two dynamometric posturographic clogs (with a total of 12 strain gauges). Participants stood upright barefoot on the clogs, in a conventional manner (feet side by side, forming an angle of 30° with both heels separated by 4 cm), with their arms alongside the body. To ensure participants security, the platform was settled in the middle of handlebars and an evaluator stood behind the participants to avoid them falling. The examination took place in a dedicated quiet room with standardized lighting conditions. Assessments were alternatively performed in ST (posture only, 4 trials) and in DT (posture and Stroop test performed simultaneously, 4 trials). Each trial lasted 30 s and a minimal 30 s-period of rest sitting was systematic between trials. In ST, subjects were instructed to maintain their balance while looking straight-ahead at a fixed red sight displayed on the screen installed 1.5 m ahead of each participant. In DT, subjects were asked to maintain the erect posture as still as possible and to perform the Stroop test at the best of their capacity without any task prioritization. Data were recorded with a sampling rate of 40 Hz and calculated using the Posturewin 4 software. As a marker of an efficient standing postural control, the amount of sway was assessed by calculating the center of pressure (CoP) area (90% confidence ellipse, mm^2^). The smaller the area, the better the postural control^48^. Beyond center of pressure (CoP) Area, the following postural kinetic parameters were recorded and calculated: mediolateral instability, defined as one standard deviation of the CoP displacement along the mediolateral axis, anteroposterior instability, defined as one standard deviation of the CoP displacement along the anteroposterior axis and mean speed of CoP displacement (mm.s^−1^).

#### Treadmill dual-task gait and cerebral oxygenation

To investigate the link between brain oxygenation and cognitive and gait performances, we designed a DT gait test on a treadmill (Gait Trainer 3, Biodex Medical System, NY, USA), with continuous recording of cerebral oxygenation using bilateral fNIRS on the two prefrontal cortices. fNIRS is a non-invasive, optical neuroimaging technique based on neurovascular coupling to infer changes in neuronal activity^49^. fNIRS provides reproducible measurements for investigating functional activation of the human cerebral cortex by tracking changes in cerebral O_2_ status while performing cognitive tasks or walking^50^.

Left and right pre-frontal cortices hemodynamics were assessed respectively between Fp1 and F3 (left prefrontal cortex) and Fp2 and F4 (right prefrontal cortex) locations according to the international 10–20 EEG system with a 3.5-cm inter-optodes distance. The probe holders were secured to the skin with double-sided tape and maintained with Velcro headbands. Oxyhemoglobin ([HbO_2_]) and deoxyhemoglobin ([HHb]) concentration changes and the tissue saturation index (TSI) were measured throughout the testing sessions using a two-wavelength (780 and 850 nm) multichannel, continuous wave NIRS system (Oxymon MkIII, Artinis Medical Systems, the Netherlands). Total hemoglobin concentration ([HbTot]) was calculated as the sum of [HbO_2_] and [HHb] and reflects changes in tissue blood volume within the illuminated area. Data were recorded continuously at 10 Hz and filtered with a 1-s width moving Gaussian smoothing algorithm before analysis. Each measurement was visually and manually checked by a trained evaluator and only valid recordings (with at least > 90% of valid signal after having removed artefacts) were kept for final analysis.

Following a 10-min period of habituation to the treadmill^51^, each participant’s preferred walking speed was determined according to a standardized protocol (updated from^52^): participants were instructed to walk on the treadmill at an initial speed of 1.5 km.h^−1^. Speed was progressively increased manually by the investigator in increments of 0.2 .km h^−1^ every 30 s until subjects reported that they were walking at their preferred walking speed. Then 1 km.h^−1^ was added to the current speed, followed by a decrease of 0.2 km.h^−1^ to confirm preferred walking speed. This procedure was repeated until a ± 0.4 km.h^−1^ agreement was obtained in preferred walking speed, as recommended^52^. The same speed was retained for the post-intervention evaluation.

The DT gait evaluation protocol was divided in 3 consecutive phases (Supplementary Figure S1): (1) Standing phase composed of 5 min of quiet standing immediately followed by 2 different conditions of cognitive assessment performed in ST (cognition only, baseline cognitive performance) and ending with 3 min of rest. The two different cognitive assessments were: a serial-7 test (S-7 test), consisting in subtracting 7 from a random 3-digit number and a Stroop test (performed according to the methodology previously described^10^). Each cognitive test consisted in 3 consecutive 1-min blocks interspersed by 1-min rest periods. CRR accounted for cognitive performance in ST and in DT; (2) Walking phase composed of 5 min of walk in ST (gait only, baseline gait performance) immediately followed by the 2 cognitive assessments (S-7 test and Stroop test) performed in DT (gait and cognition) and ending with 3 min of walk in ST. In ST gait, participants were instructed to walk according to their natural pattern, arms moving freely by their sides, while looking straight-ahead at a fixed red sight displayed on the screen. In DT, subjects were asked to walk as naturally as possible and to perform the S-7 test or Stroop test at the best of their capacity without any task prioritization; (3) Recovery phase consisting in 5 min of quiet standing. The DT gait evaluation protocol is further illustrated in Supplementary Figure S1.

### Data and statistical analysis

Study design and data are reported in accordance with the Consolidated Standards of Reporting Trials (CONSORT) criteria^53^. Patients were randomized by an independent statistician according to a computer-generated list following a 1:1 ratio. Data were analyzed in complete case analysis. Except where clearly stated, results are presented as Median [Quartile 1 (Q1); Quartile 3 (Q3)]. All data have been tested for normality prior to further analysis. For non-normally distributed data, a logarithmic transformation has been applied. Baseline data were compared (CPAP vs*.* sham-CPAP) by a non-parametric Mann–Whitney test for continuous variables, and χ^2^ test or Fisher exact test for categorical variables. For the analysis of data evolution between baseline (pre) and eight weeks (post-intervention), a linear mixed effect model was used with a patient random effect. Group effects (CPAP vs*.* sham-CPAP) and period effects (pre vs*.* post CPAP/sham-CPAP) were added as fixed effects. Interaction terms (Group * Period) were tested for all variables, but no significant effect was observed. Therefore, interaction terms were not included in the final model. For complete information of the readers, results of the linear mixed effect model with interaction terms are displayed in Supplementary Tables S4 and S5. Deltas (Pre-Post intervention in Sham-CPAP and CPAP group) for primary outcome were also calculated and between group comparisons using independent sample t-tests were performed. Results are displayed in Supplementary Table S6.

A *p* value < 0.05 was considered significant. Sample size calculation was based on a previous study from our group^10^. The sample size needed to observe a significant difference in STV of 0.8, with a standard deviation of 0.6, a power of 80% and an α value of 0.05 was 10 patients per arm. Moreover, we expected that approximatively 20% of OSAS patients would not be compliant with CPAP therapy. Consequently, we estimated that 12 patients in each group would be sufficient to show differences before and after effective or sham-CPAP therapy. Statistical analyses were performed using SAS (V.9.4, SAS Institute, Cary, NC, USA).

## Supplementary information

Supplementary information.

## Data Availability

Study protocol as well as data collected for the study, including deidentified individual participant data will be made available to others following the publication of this article, for academic purposes (e.g. meta-analyzes) on request to the corresponding author.
