# Supplementary material for: A randomized sham-controlled trial on the effect of continuous positive airway pressure treatment on gait control in severe obstructive sleep apnea patients
Source: Sci Rep. 2021 Apr 29;11:9329. doi: 10.1038/s41598-021-88642-5 (PMC8085224; doi:10.1038/s41598-021-88642-5)

**Supplementary information**

**-**

**A randomized sham-controlled trial on the effect of continuous positive airway pressure treatment on gait control in severe obstructive sleep apnea patients.**

Sébastien Baillieul^1,2^ MD, PhD, Bernard Wuyam^1,2^ MD, PhD, Dominic Pérennou^3,4^ MD, PhD Renaud Tamisier^1,2^ MD, PhD, Sébastien Bailly^1,2^ PharmD, PhD, Meriem Benmerad^1,2^ MSc, Céline Piscicelli^3,4^ PhD, Thibault Le Roux-Mallouf^1,2^ PhD, Samuel Vergès^1,2,#^ PhD, Jean-Louis Pépin^1,2,#,*^ MD, PhD

^#^ SV and JLP are joint senior authors as they contributed equally to the manuscript.

* Corresponding author

**Affiliations:**

^1^ HP2 laboratory, U1300, INSERM, Grenoble Alpes University, Grenoble, France

^2^ Pôle Thorax et Vaisseaux, Grenoble Alpes University Hospital, Grenoble, France

^3^ LPNC laboratory (UMPR CNRS 5105), Grenoble Alpes University, Grenoble, France

^4^ PMR Department, Grenoble Alpes University Hospital, Grenoble, France

**Supplementary Table S1.** Gait parameters and Stroop test performance at baseline and after 8 weeks of CPAP or Sham-CPAP.

**Supplementary Table S2.** Postural kinetic parameters and Stroop test performance at baseline and their evolution following intervention.

**Supplementary Table S3.** Cognitive performances in single (cognitive task only) and dual task (cognitive task while walking) during the treadmill test, at baseline and their evolution following intervention.

**Supplementary Table S4.** Gait parameters and Stroop test performance at baseline and after 8 weeks of CPAP or Sham-CPAP. Results of the linear mixed effect model including tests for interaction.

**Supplementary Table S5.** Postural kinetic parameters and Stroop test performance at baseline and their evolution following intervention. Results of the linear mixed effect model including tests for interaction.

**Supplementary Table S6.** Delta in primary outcome-related gait parameters and Stroop test performance between baseline and after 8 weeks of CPAP or Sham-CPAP.

**Supplementary Figure S1.** Treadmill gait assessment with parallel assessment of prefrontal cortices oxygenation using functional Near Infrared Spectroscopy.

This supplementary material has been provided by the authors to give readers additional information about their work.

**Supplementary Table S1.** Gait parameters and Stroop test performance at baseline and after 8 weeks of CPAP or Sham-CPAP.

| **Gait and cognitive parameters** | **CPAP** | | **Sham-CPAP** | | **Effect** | | | |  |
| --- | --- | --- | --- | --- | --- | --- | --- | --- | --- |
|  | **Pre** | **Post** | **Pre** | **Post** | **Group**  **(Ref.=Sham-CPAP)** | | **Period**  **(Ref.=Pre)** | |  |
|  | **Median [Q1; Q3]** | **Median [Q1; Q3]** | **Median [Q1; Q3]** | **Median [Q1; Q3]** | **β (Se)** | ***p*** | **β (Se)** | ***p*** |  |
| **STV** | | | | | | | | | |
| ST | 2.92 [2.38; 3.61] | 2.35 [2.12; 3.15] | 2.46 [2.04; 2.81] | 2.22 [1.94; 2.79] | 0.46 (0.31) | 0.14 | -0.09 (0.13) | 0.50 |  |
| DT | 2.85 [2.52; 3.24] | 2.67 [2.47; 2.89] | 2.36 [2.04; 3.22] | 2.06 [1.97; 2.69] | 0.45 (0.38) | 0.25 | -0.26 (0.09) | **<.01** |  |
| DTE | 16.5 [7.98; 21.06] | 9.54 [3.63; 16.36] | 19.59 [9.29; 28.62] | 10.27 [5.76; 17.79] | -1.95 (3.23) | 0.55 | -6.3 (3.19) | 0.06 |  |
| **Speed (m^.^s^-1^)** | | | | | | | | | |
| ST | 1.08 [1.03; 1.16] | 1.07 [1.05; 1.12] | 1.1 [1.02; 1.26] | 1.21 [1.07; 1.28] | -0.06 (0.06) | 0.31 | 0.03 (0.02) | 0.15 |  |
| DT | 1.01 [0.94; 1.09] | 1.04 [1.00; 1.07] | 1.06 [1.01; 1.22] | 1.18 [1.04; 1.28] | -0.09 (0.07) | 0.20 | 0.04 (0.02) | **0.01** |  |
| DTE | 4.99 [1.80; 9.19] | 4.05 [2.26; 5.98] | 3.12 [1.44; 5.91] | 2.05 [1.06; 3.41] | 2.53 (1.45) | 0.09 | -0.68 (0.45) | 0.14 |  |
| **Cadence (step^.^s^-1^)** | | | | | | | | | |
| ST | 110.04 [107.57; 114.08] | 109.13 [107.40; 114.57] | 106.98 [102.91; 115.96] | 112.45 [104.76; 117.95] | 0.22 (3.01) | 0.94 | 1.79 (0.76) | **0.03** |  |
| DT | 107.45 [104.73; 112.11] | 108.27 [106.44; 111.43] | 105.77 [102.85; 112.84] | 110.31 [104.91; 116.93] | -1.76 (3.17) | 0.58 | 2.03 (0.68) | **<.01** |  |
| DTE | 1.78 [0.63; 4.89] | 1.46 [0.79; 4.32] | 1.28 [0.89; 2.60] | 1.22 [0.77; 1.91] | 0.96 (0.8) | 0.25 | -0.06 (0.17) | 0.72 |  |
| **Stride time (s)** | | | | | | | | | |
| ST | 1.09 [1.06; 1.12] | 1.10 [1.05; 1.12] | 1.13 [1.04; 1.17] | 1.07 [1.02; 1.15] | 0.00 (0.03) | 0.92 | -0.02 (0.01) | **0.03** |  |
| DT | 1.12 [1.08; 1.15] | 1.11 [1.09; 1.13] | 1.14 [1.07; 1.17] | 1.09 [1.03; 1.15] | 0.01 (0.03) | 0.65 | -0.02 (0.01) | **<.01** |  |
| DTE | 1.79 [0.47; 4.76] | 1.45 [0.78; 4.44] | 1.29 [0.80; 2.64] | 1.33 [0.73; 1.84] | 0.96 (0.84) | 0.26 | -0.12 (0.17) | 0.49 |  |
| **Total double support (% total gait cycle time)** | | | | | | | | | |
| ST | 31.01 [29.83; 32.87] | 31.31 [29.43; 32.01] | 29.13 [27.53; 31.41] | 28 [26.73; 29.71] | 1.87 (1.15) | 0.12 | -0.41 (0.29) | 0.18 |  |
| DT | 31.89 [30.88; 34.02] | 31.96 [30.71; 32.67] | 29.62 [27.75; 31.92] | 28.08 [27.34; 30.8] | 2.31 (1.28) | 0.09 | -0.74 (0.28) | **0.02** |  |
| DTE | 3.30 [2.11; 5.58] | 3.37 [2.40; 3.9] | 2.38 [0.76; 3.28] | 0.73 [0.24; 2.46] | 1.68 (0.89) | 0.07 | -0.60 (0.4) | 0.15 |  |
| **Step width (cm)** | | | | | | | | | |
| ST | 14.47 [11.79; 15.13] | 14.39 [13.17; 15.9] | 12.85 [12.41; 13.66] | 13.55 [12.56; 14.89] | 0.15 (0.88) | 0.87 | 0.00 (0.26) | 0.99 |  |
| DT | 13.97 [11.5; 15.39] | 13.63 [13.22; 16.06] | 13.06 [12.60; 14.15] | 13.62 [12.92; 14.31] | 0.01 (1.01) | 0.99 | -0.07 (0.19) | 0.73 |  |
| DTE | 4.46 [1.86; 6.11] | 4.50 [1.45; 10.22] | 4.50 [1.51; 6.50] | 3.25 [1.14; 8.81] | 0.27 (1.25) | 0.83 | 0.70 (0.89) | 0.44 |  |
| **Walk ratio (cm/(steps/min))*** | | | | | | | | | |
| ST | 0.56 [0.52; 0.58] | 0.56 [0.49; 0.57] | 0.57 [0.55; 0.60] | 0.55 [0.55; 0.59] | -0.03 (0.02) | 0.26 | 0.00 (0.00) | 0.39 |  |
| DT | 0.56 [0.51; 0.59] | 0.56 [0.51; 0.6] | 0.57 [0.55; 0.59] | 0.57 [0.54; 0.59] | -0.02 (0.02) | 0.34 | 0.00 (0.00) | 0.81 |  |
| DTE | 2.46 [1.55; 3.35] | 3.68 [1.72; 4.31] | 0.87 [0.43; 2.7] | 1.28 [0.31; 2.91] | 0.91 (0.63) | 0.16 | 0.21 (0.45) | 0.65 |  |
| **Stroop test CRR** | | | | | | | | | |
| ST | 74.97 [67.73; 90.39] | 98.04 [89.29; 101.05] | 82.16 [60.65; 103.30] | 82.64 [68.49; 107.53] | 1.23 (9.13) | 0.89 | 10.31 (3.64) | **0.01** |  |
| DT | 87.45 [83.92; 95.14] | 102.88 [95.76; 112.46] | 86.50 [80.62; 98.57] | 93.32 [88.80; 101.00] | 2.00 (5.05) | 0.70 | 7.86 (1.78) | **<.01** |  |
| DTE | 20.50 [9.73; 28.85] | 9.41 [5.88; 15.96] | 14.96 [6.04; 45.63] | 15.2 [9.57; 59.00] | -13.77 (8.30) | 0.11 | -4.79 (5.79) | 0.42 |  |

**Abbreviations:** CPAP: Continuous positive airway pressure; CRR: Correct Response Rate; DT: dual task; DTE: dual task effect [DTE=(DT gait performance-ST gait performance)/ST gait performance*100]; Q1: 1^st^ quartile; Q3: 3^rd^ quartile; Ref.: reference; ST: single task; STV: stride time variability.

Data are presented as median [Q1; Q3]. Analysis: a linear mixed effect model was performed with a patient random effect. Group effects (CPAP vs. Sham-CPAP) and period effects (pre vs. post CPAP/Sham-CPAP) were added as fixed effects. Significant results are displayed in bold type.

*Walk ratio (WR): WR=Step Length (cm)/cadence (steps/min). WR is a speed-independent index of the overall neuromotor gait control, which reflects balance, between-step

variability, and attentional demand.

**Supplementary Table S2.** Postural kinetic parameters and Stroop test performance at baseline and their evolution following intervention.

| **Postural kinetic and cognitive parameters** | **CPAP** | | **Sham-CPAP** | | **Effect** | | | |
| --- | --- | --- | --- | --- | --- | --- | --- | --- |
|  | **Pre** | **Post** | **Pre** | **Post** | **Group (Ref.=Sham-CPAP)** | | **Period (Ref.=Pre)** | |
|  | **Median [Q1; Q3]** | **Median [Q1; Q3]** | **Median [Q1; Q3]** | **Median [Q1; Q3]** | **β (Se)** | ***p*** | **β (Se)** | ***p*** |
| **CoP Area (mm^2^)** | | | | | | | | |
| ST | 84.78 [53.72; 100.47] | 65.16 [47.73; 116.15] | 96.4 [67.86; 138.92] | 85.9 [50.86; 137.35] | -15.48 (27.88) | 0.58 | 0.37 (17.36) | 0.98 |
| DT | 98.14 [47.89; 112.28] | 74.37 [56.34; 141.00] | 104.58 [61.28; 118.78] | 110.44 [80.82; 181.11] | -16.85 (22.60) | 0.46 | 26.22 (15.59) | 0.11 |
| DTE | 31.77 [18.71; 60.58] | 37.91 [10.4; 51.34] | 44.47 [21.72; 60.67] | 43.22 [17.47; 102.85] | -15.04 (11.55) | 0.21 | 5.31 (10.80) | 0.63 |
| **Mediolateral instability (One SD of CoP displacement length (mm))** | | | | | | | | |
| ST | 1.79 [1.38; 2.05] | 1.69 [1.24; 2.05] | 1.75 [1.43; 2.12] | 2.00 [1.26; 2.26] | -0.23 (0.32) | 0.48 | -0.05 (0.19) | 0.80 |
| DT | 1.77 [1.18; 2.33] | 1.85 [1.54; 2.12] | 2.08 [1.36; 2.43] | 1.96 [1.79; 2.99] | -0.22 (0.3) | 0.47 | 0.19 (0.19) | 0.32 |
| DTE | 21.62 [11.12; 34.64] | 26.16 [14.43; 32.54] | 25.58 [9.44; 37.14] | 21.53 [9.24; 65.62] | -4.33 (9.04) | 0.64 | 5.33 (6.91) | 0.45 |
| **Anteroposterior instability (One SD of CoP displacement length (mm))** | | | | | | | | |
| ST | 3.24 [2.80; 4.32] | 3.39 [2.68; 4.06] | 3.72 [2.99; 4.64] | 3.51 [3.05; 4.92] | -0.18 (0.46) | 0.70 | 0.06 (0.22) | 0.77 |
| DT | 3.65 [2.96; 3.74] | 3.76 [2.67; 4.77] | 3.34 [3.14; 4.45] | 3.75 [3.06; 4.63] | -0.29 (0.40) | 0.47 | 0.37 (0.25) | 0.15 |
| DTE | 15.5 [7.90; 25.55] | 18.8 [12.80; 20.16] | 16.56 [6.73; 36.98] | 20.28 [7.68; 33.35] | -5.25 (5.74) | 0.37 | -0.14 (4.45) | 0.97 |
| **Mean speed (mm^.^s^-1^)** | | | | | | | | |
| ST | 7.58 [6.63; 9.12] | 8.43 [6.59; 9.06] | 8.68 [7.39; 9.51] | 9.22 [7.17; 9.63] | -0.45 (0.79) | 0.57 | 0.39 (0.36) | 0.30 |
| DT | 9.82 [8.45; 11.74] | 9.65 [9.40; 10.99] | 9.65 [7.71; 11.92] | 10.39 [8.23; 12.85] | -0.22 (1.10) | 0.84 | 0.39 (0.40) | 0.34 |
| DTE | 26.88 [13.19; 43.88] | 21.38 [8.95; 32.27] | 22.76 [3.89; 30.01] | 16.3 [8.88; 52.04] | 2.8 (8.76) | 0.75 | 0.79 (4.34) | 0.86 |
| **Stroop test CRR** | | | | | | | | |
| ST | 74.97 [67.73; 90.39] | 98.04 [89.29; 101.05] | 82.16 [60.65; 103.3] | 82.64 [68.49; 107.53] | 1.23 (9.13) | 0.89 | 10.31 (3.64) | **0.01** |
| DT | 78.33 [69.44; 82.78] | 83.33 [78.89; 88.89] | 75 [63.89; 85.00] | 77.78 [73.89; 80.56] | 2.62 (4.32) | 0.55 | 6.42 (1.71) | **<.01** |
| DTE | 8.42 [3.03; 16.57] | 12.89 [9.04; 18.40] | 26.56 [11.93; 30.89] | 27.05 [16.64; 32.43] | -13.75 (4.32) | **<.01** | 1.41 (3.46) | 0.69 |

**Abbreviations:** CoP: center of pressure; CPAP: Continuous positive airway pressure; CRR: Correct Response Rate; DT: dual task; DTE: dual task effect [DTE= ∣(DT postural performance-ST postural performance)/ST postural performance*100∣ ]; Q1: 1^st^ quartile; Q3: 3^rd^ quartile; Ref.: reference; SD: standard deviation; ST: single task.

Data are presented as median [Q1; Q3]. Analysis: linear mixed effect model was performed with a patient random effect. Group effects (CPAP vs. Sham-CPAP) and period effects (pre vs. post CPAP/Sham-CPAP) were added as fixed effects. Significant results are displayed in bold.

**Supplementary Table S3.** Cognitive performances in single (cognitive task only) and dual task (cognitive task while walking) during the treadmill test, at baseline and their evolution following intervention.

| **Cognitive performances** | **CPAP** | | **sham-CPAP** | | **Effect** | | | |
| --- | --- | --- | --- | --- | --- | --- | --- | --- |
|  | **Pre** | **Post** | **Pre** | **Post** | **Group**  **(Ref.=Sham-CPAP)** | | **Period**  **(Ref.=Pre)** | |
|  | **Median [Q1; Q3]** | **Median [Q1; Q3]** | **Median [Q1; Q3]** | **Median [Q1; Q3]** | **β (Se)** | ***p*** | **β (Se)** | ***p*** |
| FIRST TRIAL ONLY | | | | | | | | |
| **S-7 CRR** | | | | | | | | |
| ST | 9.15 [5.85; 15.85] | 8.30 [5.00; 13.30] | 13.30 [6.65; 16.70] | 16.7 [10.80; 20.85] | -1.14 (4.71) | 0.81 | 1.92 (1.14) | 0.11 |
| DT | 15.00 [6.70; 23.30] | 11.70 [6.70; 20.00] | 15.85 [9.15 ; 22.5] | 14.15 [9.15 ; 27.50] | 0.09 (5.34) | 0.99 | 1.20 (1.27) | 0.36 |
| DTE | 19.28 [0.00; 50.00] | 87.22 [20.12; 123.88] | 29.07 [14.41 ; 36.76] | 27.71 [4.79; 54.18] | 16.24 (14.43) | 0.27 | 13.62 (15.87) | 0.40 |
| **Stroop test CRR** | | | | | | | | |
| ST | 73.30 [70.00; 79.20] | 85.00 [71.70; 91.70] | 66.65 [61.70; 86.65] | 75.85 [69.20; 84.15] | 2.80 (4.81) | 0.57 | 5.29 (1.76) | **<.01** |
| DT | 75.00 [66.70; 82.50] | 86.70 [81.70; 90.00] | 75.80 [70.00; 84.15] | 75 [67.50; 79.20] | 3.60 (4.43) | 0.42 | 3.59 (1.79) | 0.06 |
| DTE | 4.35 [2.85; 9.17] | 5.36 [0.00; 9.66] | 11.92 [4.91; 16.67] | 4.41 [0.86; 8.12] | -1.49 (2.41) | 0.54 | -5.03 (1.85) | **0.01** |
| MEAN PERFORMANCE FOR THE THREE TRIALS | | | | | | | | |
| **S-7 CRR** | | | | | | | | |
| ST | 11.10 [7.22; 18.07] | 10.57 [8.33; 12.23] | 14.43 [8.60; 20.57] | 15.55 [10.27; 19.73] | -0.82 (4.46) | 0.86 | 1.42 (0.86) | 0.12 |
| DT | 12.22 [7.23; 19.72] | 14.43 [7.77; 15.57] | 18.35 [9.72; 23.05] | 15.00 [10.00; 29.17] | -0.45 (5.24) | 0.93 | 1.12 (1.10) | 0.32 |
| DTE | 24.30 [14.94; 33.40] | 29.64 [24.93; 55.33] | 17.80 [11.02; 30.84] | 23.22 [12.51; 42.38] | 5.90 (8.26) | 0.48 | 12.12 (8.40) | 0.16 |
| **Stroop test CRR** | | | | | | | | |
| ST | 73.88 [69.18; 80.27] | 87.77 [75.03; 88.90] | 68.05 [60.02; 85.02] | 76.12 [67.50; 84.70] | 4.01 (4.54) | 0.39 | 5.30 (1.41) | **<.01** |
| DT | 75.27 [70.55; 82.23] | 86.67 [81.10; 93.33] | 72.5 [66.38; 83.60] | 75.00 [69.43; 83.90] | 3.87 (4.33) | 0.38 | 4.93 (1.29) | **<.01** |
| DTE | 2.32 [1.55; 3.95] | 4.39 [2.36; 5.17] | 5.31 [3.38; 10.85] | 2.15 [1.10; 6.03] | -1.28 (1.41) | 0.37 | -1.57 (1.24) | 0.22 |

**Abbreviations:** CPAP: Continuous positive airway pressure; CRR: Correct Response Rate; DT: dual task; DTE: dual task effect [DTE=(DT gait performance-ST gait performance)/ST gait performance*100]; Q1: 1^st^ quartile; Q3: 3^rd^ quartile; Ref.: reference; S-7: Serial S-7 test; ST: single task.

Data are presented as median [Q1; Q3]. Analysis: linear mixed effect model was performed with a patient random effect. Group effects (CPAP vs. Sham-CPAP) and period effects (pre vs. post CPAP/Sham-CPAP) were added as fixed effects. Significant results are displayed in bold.

**Supplementary Table S4.** Gait parameters and Stroop test performance at baseline and after 8 weeks of CPAP or Sham-CPAP. Results of the linear mixed effect model including tests for interaction

| **Gait and cognitive parameters** | **Effect** | | | | | |
| --- | --- | --- | --- | --- | --- | --- |
|  | **Group**  **(Ref.=Sham-CPAP)** | | **Period**  **(Ref.=Pre)** | | **Interaction**  **Group*Period** | |
|  | **ß (Se)** | ***p*** | **ß (Se)** | ***p*** | **ß (Se)** | ***p*** |
| **STV** | | | | | | |
| ST | 0.53 (0.33) | 0.12 | -0.03 (0.18) | 0.87 | -0.14 (0.28) | 0.61 |
| DT | 0.46 (0.39) | 0.25 | -0.25 (0.12) | 0.05 | -0.02 (0.18) | 0.91 |
| DTE | -1.55 (4.41) | 0.73 | -5.9 (4.42) | 0.20 | -0.86 (6.53) | 0.90 |
| **Speed (m^.^s^-1^)** | | | | | | |
| ST | -0.05 (0.06) | 0.44 | 0.04 (0.02) | 0.11 | -0.03 (0.04) | 0.41 |
| DT | -0.08 (0.07) | 0.28 | 0.06 (0.02) | **0.02** | -0.03 (0.03) | 0.37 |
| DTE | 2.34 (1.51) | 0.13 | -0.88 (0.6) | 0.16 | 0.45 (0.92) | 0.63 |
| **Cadence (step^.^s^-1^)** | | | | | | |
| ST | 1.15 (3.07) | 0.71 | 2.72 (0.98) | **0.01** | -2.15 (1.5) | 0.17 |
| DT | -0.81 (3.21) | 0.80 | 2.99 (0.87) | **<.01** | -2.21 (1.32) | 0.11 |
| DTE | 0.96 (0.82) | 0.25 | -0.06 (0.24) | 0.79 | 0 (0.36) | 1.00 |
| **Stride time (s)** | | | | | | |
| ST | -0.01 (0.03) | 0.70 | -0.03 (0.01) | **0.01** | 0.02 (0.01) | 0.19 |
| DT | 0.01 (0.03) | 0.86 | -0.03 (0.01) | **<.01** | 0.02 (0.01) | 0.14 |
| DTE | 0.95 (0.85) | 0.27 | -0.13 (0.23) | 0.58 | 0.03 (0.35) | 0.93 |
| **Total double support (% total gait cycle time)** | | | | | | |
| ST | 1.67 (1.18) | 0.17 | -0.61 (0.39) | 0.13 | 0.47 (0.59) | 0.43 |
| DT | 2.04 (1.31) | 0.13 | -1 (0.37) | **0.01** | 0.62 (0.57) | 0.29 |
| DTE | 1.31 (0.96) | 0.18 | -0.97 (0.54) | 0.09 | 0.84 (0.81) | 0.31 |
| **Step width (cm)** | | | | | | |
| ST | 0.41 (0.92) | 0.66 | 0.26 (0.33) | 0.44 | -0.6 (0.51) | 0.25 |
| DT | 0.1 (1.03) | 0.92 | 0.02 (0.26) | 0.95 | -0.2 (0.39) | 0.62 |
| DTE | 0.16 (1.49) | 0.91 | 0.59 (1.23) | 0.64 | 0.24 (1.84) | 0.90 |
| **Walk ratio (cm/(steps/min)) *** | | | | | | |
| ST | -0.03 (0.02) | 0.22 | -0.01 (0.01) | 0.30 | 0.01 (0.01) | 0.53 |
| DT | -0.03 (0.02) | 0.29 | 0 (0.01) | 0.77 | 0.01 (0.01) | 0.48 |
| DTE | 0.46 (0.74) | 0.54 | -0.25 (0.6) | 0.68 | 1.01 (0.9) | 0.27 |
| **CRR** | | | | | | |
| ST | -3.11 (9.61) | 0.75 | 5.62 (4.78) | 0.25 | 10.29 (7.1) | 0.16 |
| DT | 0.4 (5.26) | 0.94 | 6.26 (2.37) | **0.02** | 3.69 (3.6) | 0.32 |
| DTE | -10.05 (9.72) | 0.31 | -0.79 (8) | 0.92 | -8.58 (11.79) | 0.47 |

**Abbreviations:** CPAP: Continuous positive airway pressure; CRR: Correct Response Rate; DT: dual task; DTE: dual task effect [DTE=(DT gait performance-ST gait performance)/ST gait performance*100]; Ref.: reference; ST: single task; STV: stride time variability.

Analysis: a linear mixed effect model was performed with a patient random effect. Group effects (CPAP vs. Sham-CPAP) and period effects (pre vs. post CPAP/Sham-CPAP) were added as fixed effects and interaction (Group*Period) were tested. Significant results are displayed in bold type.

*Walk ratio (WR): WR=Step Length (cm)/cadence (steps/min). WR is a speed-independent index of the overall neuromotor gait control, which reflects balance, between-step

variability, and attentional demand.

**Supplementary Table S5.** Postural kinetic parameters and Stroop test performance at baseline and their evolution following intervention. Results of the linear mixed effect model including tests for interaction

| **Postural kinetic and cognitive parameters** | **Effect** | | | | | |
| --- | --- | --- | --- | --- | --- | --- |
|  | **Group**  **(Ref.=Sham-CPAP)** | | **Group**  **(Ref.=Sham-CPAP)** | | **Group**  **(Ref.=Sham-CPAP)** | |
|  | **ß (Se)** | **ß (Se)** | **ß (Se)** | **ß (Se)** | **ß (Se)** | **ß (Se)** |
| **CoP Area (mm^2^)** | | | | | | |
| ST | -21.37 (32.05) | 0.51 | -5.52 (23.74) | 0.82 | 13.24 (35.69) | 0.71 |
| DT | -4.72 (26.53) | 0.86 | 38.35 (21.2) | 0.09 | -27.28 (31.78) | 0.40 |
| DTE | -4.66 (15.16) | 0.76 | 15.69 (14.67) | 0.30 | -22.64 (21.74) | 0.31 |
| **Mediolateral instability (One SD of CoP displacement length (mm))** | | | | | | |
| ST | -16.88 (10.42) | 0.12 | -4.42 (6.9) | 0.53 | 9.09 (10.42) | 0.39 |
| DT | 8.24 (16.59) | 0.62 | 8.38 (6.78) | 0.23 | -9.66 (10.32) | 0.36 |
| DTE | 20.09 (14.5) | 0.18 | 5.56 (5.43) | 0.32 | -9.57 (8.27) | 0.26 |
| **Anteroposterior instability (One SD of CoP displacement length (mm))** | | | | | | |
| ST | 2.82 (24.23) | 0.91 | 23.39 (15.05) | 0.14 | -19.84 (22.75) | 0.39 |
| DT | 2.68 (31.28) | 0.93 | 25.57 (14.89) | 0.10 | -32.56 (22.62) | 0.16 |
| DTE | -2.04 (10.26) | 0.84 | 1.19 (6.85) | 0.86 | -1.56 (10.33) | 0.88 |
| **Mean speed (mm^.^s^-1^)** | | | | | | |
| ST | -0.28 (0.85) | 0.74 | 0.56 (0.49) | 0.27 | -0.39 (0.74) | 0.60 |
| DT | 0.29 (1.15) | 0.80 | 0.9 (0.51) | 0.10 | -1.18 (0.78) | 0.15 |
| DTE | 5.43 (9.53) | 0.57 | 3.42 (5.91) | 0.57 | -6.11 (8.95) | 0.50 |

**Abbreviations:** CoP: center of pressure; CPAP: Continuous positive airway pressure; CRR: Correct Response Rate; DT: dual task; DTE: dual task effect [DTE= ∣(DT postural performance-ST postural performance)/ST postural performance*100∣ ]; Ref.: reference; SD: standard deviation; ST: single task.

Analysis: linear mixed effect model was performed with a patient random effect. Group effects (CPAP vs. Sham-CPAP) and period effects (pre vs. post CPAP/Sham-CPAP) were added as fixed effects and interaction (Group*Period) were tested. Significant results are displayed in bold.

**Supplementary Table S6.** Delta in primary outcome-related gait parameters and Stroop test performance between baseline and after 8 weeks of CPAP or Sham-CPAP.

| **Gait and cognitive parameters** | Sham-CPAP  Mean (SD) | CPAP  Mean (SD) | *p* |
| --- | --- | --- | --- |
| **STV** | | | |
| ST | -0.03 (0.13) | -0.14 (0.26) | 0.71 |
| DT | -0.25 (0.11) | -0.28 (0.14) | 0.87 |
| DTE | -5.90 (4.33) | -7.70 (5.46) | 0.54 |
| **StrideTime\Cycle** | | | |
| **ST** | **-0.02 (0.04)** | **-0.01 (0.01)** | **0.28** |
| **DT** | **-0.03 (0.03)** | **-0.01 (0.02)** | **0.16** |
| DTE | -0.13 (0.27) | -0.10 (0.17) | 0.93 |
| **Speed** | | | |
| ST | 0.04 (0.10) | 0.01 (0.04) | 0.47 |
| DT | 0.05 (0.09) | 0.02 (0.03) | 0.40 |
| DTE | -0.87 (0.58) | -0.37 (0.71) | 0.59 |
| **CRR** | | | |
| **ST** | **5.58 (19.04)** | **15.43 (11.39)** | **0.24** |
| DT | 6.25 (7.12) | 9.50 (9.56) | 0.40 |
| **DTE** | **-2.42 (10.50)** | **-9.47 (4.21)** | **0.46** |

**Abbreviations:** CPAP: Continuous positive airway pressure; CRR: Correct Response Rate; DT: dual task; DTE: dual task effect [DTE=(DT gait performance-ST gait performance)/ST gait performance*100]; SD: Standard Deviation; ST: single task; STV: stride time variability.

Data are presented as Mean (1 Standard Deviation [SD]). Analysis: T-tests for independent samples were performed. Non-normally distributed data have been log-transformed and are displayed in red and bold type.

**Supplementary Figure S1.** **Treadmill gait assessment with parallel assessment of prefrontal cortices oxygenation using functional Near Infrared Spectroscopy.**

**a.** Schema of the experimental protocol. **b.** Experimental setting: participant walking on the treadmill in dual task, performing a Stroop test displayed on the screen. **c.** Picture of an example of fNIRS optodes placement over the prefrontal cortices and schematic depiction (red dots) of optodes’ placement according to the 10-20 International system of EEG electrode placement.

Abbreviations: DT: dual task; S-7: Serial S-7 tasks; ST: single task.


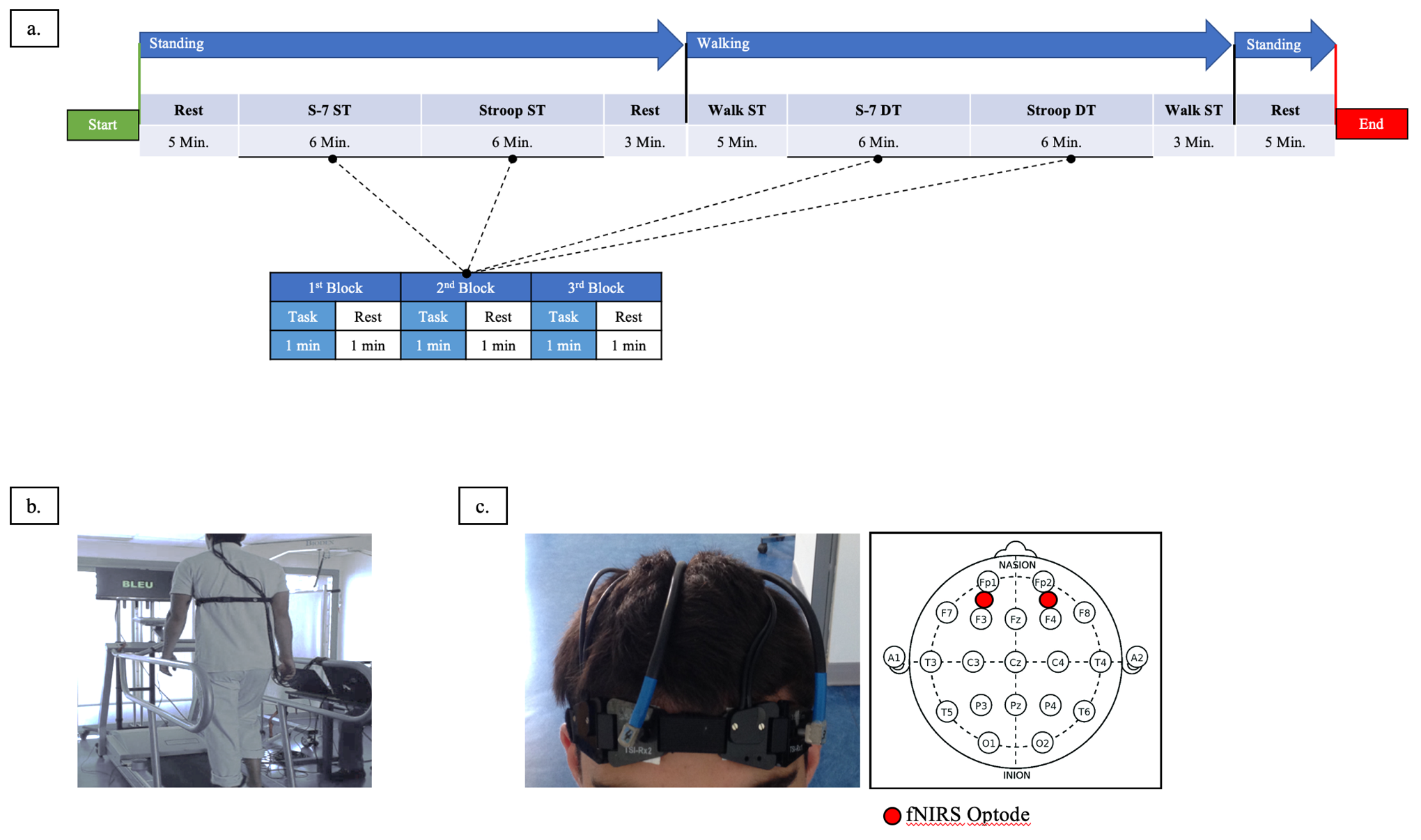

Supplement: Supplementary file 1 — Supplementary information. [file 41598_2021_88642_MOESM1_ESM.docx]
